# An interventional clinical trial investigating the effects of *Spirulina platensis* on dental fluorosis and antioxidant system in lambs reared in endemic areas

**DOI:** 10.1038/s41598-023-44058-x

**Published:** 2023-10-06

**Authors:** Abdellatif Rahim, Mounia Sibaoueih, Adekhalid Essamadi, Bouchra El Amiri

**Affiliations:** 1 Animal Production Unit, Regional Center Agricultural Research of Settat, National Institute for Agricultural Research (INRA), Avenue EnnasrRabat Principal, P.O. Box 415, 10090 Rabat, Morocco; 2 Laboratory of Biochemistry, Neurosciences, Natural Resources and Environment, Faculty of Sciences and Techniques, Hassan First University of Settat, P.O. Box 577, 26000 Settat, Morocco; 3 African Sustainable Agriculture Research Institute (ASARI), Mohammed VI Polytechnic University (UM6P), 70000 Laayoune, Morocco

**Keywords:** Environmental sciences, Natural hazards

## Abstract

This study aimed to evaluate the protective effect of *Spirulina platensis* primary against dental fluorosis and secondary against oxidative stress in lambs reared in endemic fluorosis areas. Forty-eight lambs aged 5 months were divided into four equal groups (each one including 6 males and 6 females). Groups I and II served as controls belonging respectively to fluorosis-free (Settat) and endemic fluorosis (El Fokra) areas, while the other two Groups III and IV (belonging to El Fokra) received respectively a fixed daily intake of 250 and 500 mg/kg bodyweight (BW) of *Spirulina platensis*. The experiment was carried out for 13 months until the adult incisors appeared for all animals. According to the Dean’s Fluorosis Index (DFI), 500 mg/kg BW/day of *Spirulina platensis* (Group IV) protected against dental fluorosis. Moreover, in both male and female lambs, this dose significantly (*p* < 0.0001) reduced the plasmatic levels of fluoride, proteins, GSH, and MDA compared to the Group II. Furthermore, enzymatic activities of catalase and SOD increased significantly (*p* < 0.0001) in male and female lambs of the Group IV as compared to Group II. In conclusion, our findings support the potential use of *Spirulina platensis* as a valuable solution for addressing fluorosis in sheep, warranting further clinical trials.

## Introduction

Fluorine is one of the most reactive elements in nature^1,2^. It plays an important role in the production and growth of both humans and animals^3,4^. However, chronic exposure to high fluoride level can lead to several chronic intoxications such as dental and skeletal fluorosis^5^. Another important aspect of fluoride toxicity is its impact on the antioxidant system^6^ and generation of free radicals^7^. Fluorosis is a global health problem and it is endemic in different parts of the world^8,9^. It has been documented in humans and animals, mainly ruminants^10,11^. All over the world, many regions are well known for their fluoride contamination. In certain cases, countries such as India, China, Turkey, Morocco, Mexico and Australia, sheep have gained more attention from researchers^12,13^. Furthermore, chronic ingestion of fluoride from drinking water, or from roughage growing in soils rich in fluoride or contaminated with fluorinated compounds from volcanic or industrial sources, has a negative impact on the growth and production of sheep^14,15^. This causes serious socio-economic problems for the farmers^16^.

To prevent sheep from the harmful effects of this poisoning, several strategies have been adopted either by breeders or by researchers. Since fluorosis only manifests after the appearance of the first two adult teeth in sheep, breeders commonly adopt the strategy of selling lambs before their first teeth erupt and buying adult sheep from fluorosis-free areas. This causes a loss of herd benefits due to the low selling prices of young animals and handicaps the genetic programs^17^. Besides, several technologies have been practiced in different regions of the world for the defluoridation of water^18^. However, in many cases, infrastructure requirements, technical expertise and high operating costs are major factors limiting their wide use^19,20^. Moreover, various compounds have been tested to alleviate fluorosis in sheep such as aluminum sulfate^21^, aluminum chloride^22^ and aluminum hydroxide^23^. However, chronic ingestion of these synthetic minerals could also cause a toxic effect in animals^24^. Recently, a number of reports documented the beneficial effect of natural sources of biomolecules originating from *Spirulina platensis* (microalgae) and *Tamarindus indica* L. (medicinal plant), such as bivalent minerals and antioxidants to reduce or prevent fluoride toxicity in rats, mice, rabbits and cattle^6,19,25,26^. However, all these studies investigated only experimentally induced fluorosis.

*Spirulina platensis* is known for its richness in various minerals mainly magnesium, manganese, copper, zinc, and selenium^27^, which could chelate fluoride excess and promote its excretion. In addition, this microalga contains a wide range of antioxidant including phycocyanin, carotenoids, chlorophyll, acidic polyphenols, linoleic acid and vitamin C, which could correct the oxidative stress generated by this halogen. Therefore, the present study aimed to evaluate the potential protective effect of *Spirulina platensis* at two doses (250 and 500 mg/kg BW/day) primary against dental fluorosis and secondary against oxidative stress in naturally exposed lambs in the commune of El Fokra, belonging to the province of Khouribga, Morocco, an endemic fluorosis area. The control group includs lambs reared in the fluorosis-free Settat region, considered healthy. The null hypothesis of this study was that there is no protective effect of *Spirulina platensis* at two doses (250 and 500 mg/kg BW/day) against dental fluorosis and oxidative stress in lambs.

## Results

### Fluoride levels in groundwater and soil

Table 1 presents the comparison of fluoride levels in groundwater and soil samples between El Fokra commune and the Settat area. The results revealed that fluoride levels in both water and soil samples were significantly (*p* < 0.0001) higher in El Fokra commune compared to Settat area.

**Table 1:** Comparison of fluoride levels in groundwater and soil between El Fokra commune and Settat area.

Fluoride levels (ppm)	El Fokra Commune	Settat area
Groundater	1.82 ± 0.34^A^	0.72 ± 0.09^B^
Soil	750.30 ± 32.29^A^	158.90 ± 7.66^B^

Data were expressed as the mean ± SEM.

A and B different superscripts within each column show significant differences at the level of fluoride levels between El Fokra commune and Settat area.

### Clinical examination and prevalence of dental fluorosis

Table 2 presents the results of clinical examination of dental fluorosis. According to Fisher’s exact test, the statistical results indicated that there was a significant (*p* < 0.0001) association between the supplementation of *Spirulina platensis* and the state of dentition in lambs reared in an endemic fluorosis area. Moreover, it revealed that in all studied groups, male animals had a higher incidence of dental fluorosis compared to females, however, the difference was not statistically significant (*p* > 0.05). A 100% of the females and 83% of the males reared in fluorosis-free area (Group I) had a normal score, while the rest of males (17%) had a questionable score. Moreover, female, and male animals reared in endemic fluorosis area (Group II) presented dental lesions which intensity varied from very mild to moderate scores. The 250 mg/kg BW/day intake of *Spirulina platensis* (Group III) improved the protection of the animals’ teeth with 66% of females and 50% of males presenting a normal score, 17% of females and males presenting a questionable score, and 17% of females and 34% of males with a very mild score. Finally, the 500 mg/kg BW/day intake of *Spirulina platensis* (Group IV) protected the animals’ teeth from fluorosis, with only 17% of females and 34% of males showing a questionable score, while the remaining 83% of females and 66% of males had a normal score. It has to be highlighted that Groups III and IV went only as far as very mild and questionable scores, respectively, while Group II showed mild and moderate scores.

**Table 2:** Effect of *Spirulina platensis* on the state of dentition in lambs reared in endemic fluorosis area.

Score	Group I*		Group II*		Group III*		Group IV*	
	Female	Male	Female	Male	Female	Male	Female	Male
Normal, n (%)	6 (100)	5 (83)	0	0	4 (66)	3 (50)	5 (83)	4 (66)
Questionable, n (%)	0	1 (17)	0	0	1 (17)	1 (17)	1 (17)	2 (33)
Very mild, n (%)	0	0	2 (33)	1 (17)	1 (17)	2 (33)	0	0
Mild, n (%)	0	0	3 (50)	2 (33)	0	0	0	0
Moderate, n (%)	0	0	1 (17)	3 (50)	0	0	0	0
Severe, n (%)	0	0	0	0	0	0	0	0
Total, n (%)	6 (100)	6 (100)	6 (100)	6 (100)	6 (100)	6 (100)	6 (100)	6 (100)

*Indicates significant difference in the severity the state of dentition between male and female groups (*p* < 0.0001); n: number of animals; Group I: lambs reared in fluorosis-free area, Group II: lambs reared in endemic fluorosis area, Group III: lambs supplemented with 250 mg/kg BW/day of *Spirulina platensis*, Group IV: lambs supplemented with 500 mg/kg BW/day of *Spirulina platensis*.

### Plasma fluoride level

**Figure 1:**
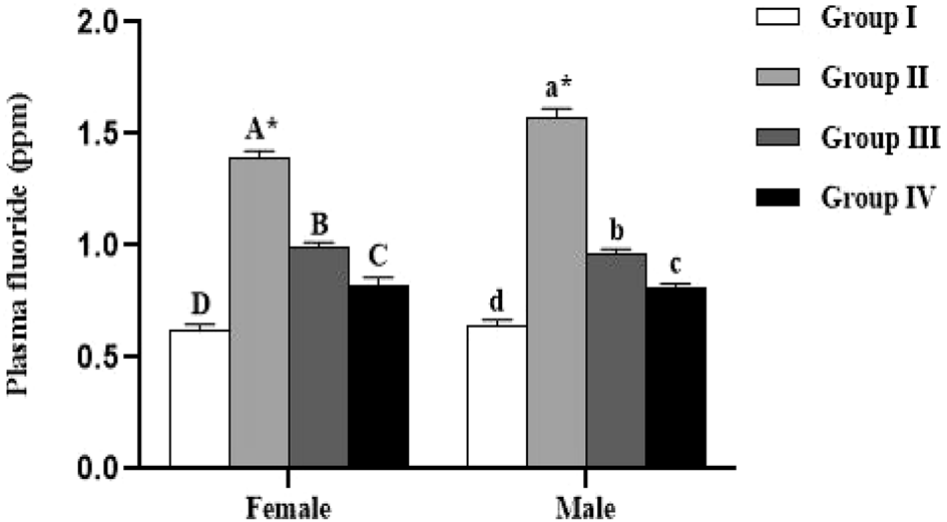
Effect of *Spirulina platensis* on plasma fluoride level in lambs reared in endemic fluorosis area. Data were expressed as the mean ± SEM. Group I: lambs reared in fluorosis-free area, Group II: lambs reared in endemic fluorosis area, Group III: lambs supplemented with 250 mg/kg BW/day of *Spirulina platensis*, Group IV: lambs supplemented with 500 mg/kg BW/day of *Spirulina platensis*. A, B, C and D different superscripts within each column show significant differences at the level of plasma fluoride between the females of each group. a, b, c and d different superscripts within each column show significant differences at the level of plasma fluoride between the males of each group. *: indicates the significant difference at the level of plasma fluoride between males and females of each group.

The results indicated that the plasma fluoride level was significantly higher in female (*p* < 0.0001) and male (*p* < 0.0001) lambs reared in endemic fluorosis area (Group II) compared to the those reared in fluorosis-free area (Group I) (Fig. 1). Furthermore, the group supplemented with 250 mg/kg BW/day (Group III) of *Spirulina platensis* revealed that the plasma fluoride level was significantly higher in females (*p* < 0.0001) and males (*p* = 0.0002) compared to the Group I and significantly lower compared to those from Group II (*p* < 0.0001) (Fig. 1). Concerning the group supplemented by 500 mg/kg BW/day (Group IV) of *Spirulina platensis* revealed that the plasma fluoride level was significantly higher in females (*p* < 0.0454) and males (*p* < 0.0023) compared to the Group I and significantly lower compared to those of Group II (*p* < 0.0001) (Fig. 1). The results also showed that the dose of 250 mg/kg BW per day resulted in a fewer effective decreases of plasma fluoride with only 51.60% in females and 65.61% in males. In contrast, 500 mg/kg of *Spirulina platensis* per day resulted in a better decrease of plasma fluoride in both male and female lambs. Specifically, the reduction in plasma fluoride was 73.15% for females and 81.28% for males.

### Fecal fluoride level

**Figure 2:**
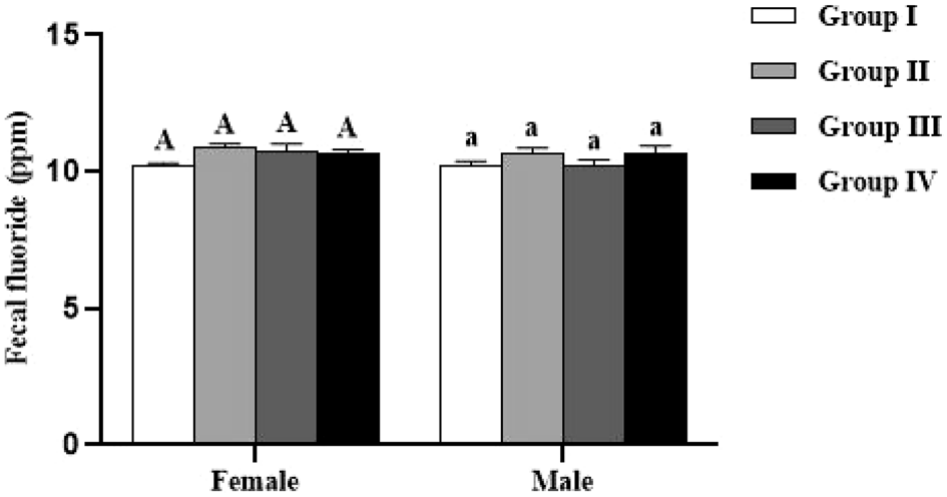
Effect of *Spirulina platensis* on fecal fluoride levels in lambs reared in endemic fluorosis area. Data were expressed as the mean ± SEM. Group I: lambs reared in fluorosis-free area, Group II: lambs reared in endemic fluorosis area, Group III: lambs supplemented with 250 mg/kg BW/day of *Spirulina platensis*, Group IV: lambs supplemented with 500 mg/kg BW/day of *Spirulina platensis*. A: indicates that there is no significant difference in fecal fluoride levels between the females of each group. a indicates that there is no significant difference in fecal fluoride levels between the males of each group.

The Fig. 2 shows the level of fluoride in the feces samples. The results indicate that the parameter under investigation was not significantly affected by either the studied groups or sex (*p* > 0.05).

### Biochemical parameters

**Figure 3:**
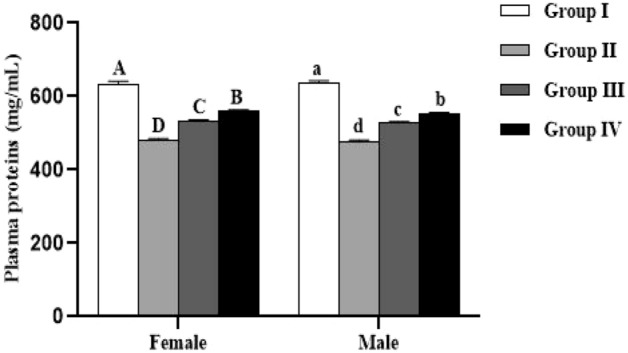
Effect of *Spirulina platensis* on plasma proteins level in lambs reared in endemic fluorosis area. Data were expressed as the mean ± SEM. Group I: lambs reared in fluorosis-free area, Group II: lambs reared in endemic fluorosis area, Group III: lambs supplemented with 250 mg/kg BW/day of *Spirulina platensis*, Group IV: lambs supplemented with 500 mg/kg BW/day of *Spirulina platensis*. A, B, C and D different superscripts within each column show significant differences at the level of plasma proteins between the females of each group. a, b, c and d different superscripts within each column show significant differences at the level of plasma proteins between the males of each group.

**Figure 4:**
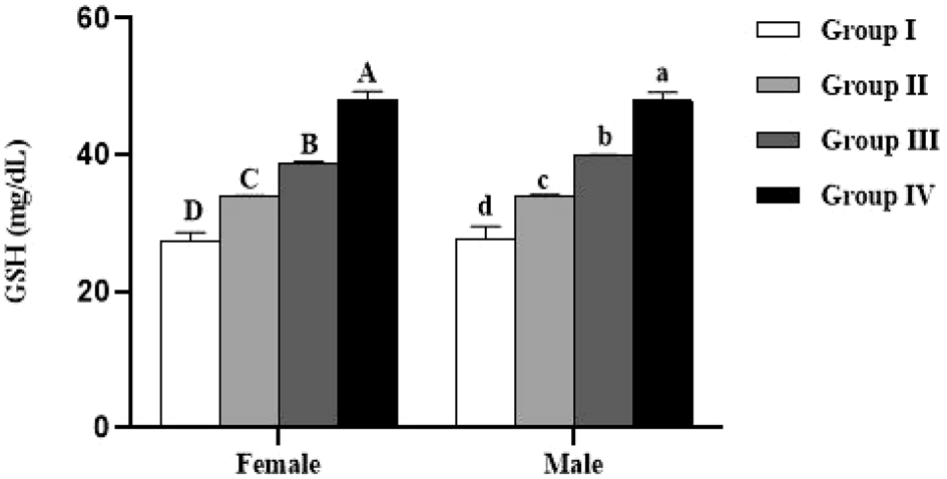
Effect of *Spirulina platensis* on reduced glutathione (GSH) level in lambs reared in endemic fluorosis area. Data were expressed as the mean ± SEM. Group I: lambs reared in fluorosis-free area, Group II: lambs reared in endemic fluorosis area, Group III: lambs supplemented with 250 mg/kg BW/day of *Spirulina platensis*, Group IV: lambs supplemented with 500 mg/kg BW/day of *Spirulina platensis*. A, B, C and D different superscripts within each column show significant differences at the level of GSH between the females of each group (*p* < 0.05). a, b, c and d different superscripts within each column show significant differences at the level of GSH between the males of each group.

**Figure 5:**
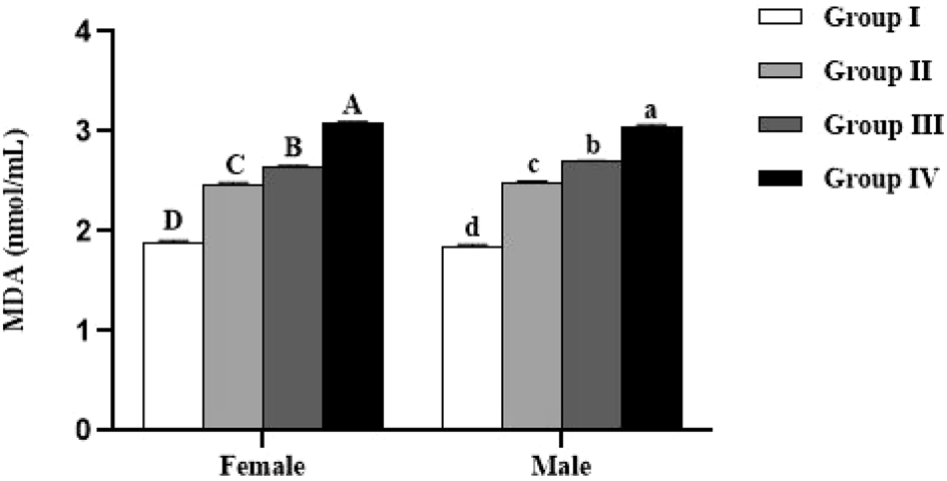
Effect of *Spirulina platensis* on malondialdehyde (MDA) level in lambs reared in endemic fluorosis area. Data were expressed as the mean ± SEM. Group I: lambs reared in fluorosis-free area, Group II: lambs reared in endemic fluorosis area, Group III: lambs supplemented with 250 mg/kg BW/day of *Spirulina platensis*, Group IV: lambs supplemented with 500 mg/kg BW/day of *Spirulina platensis*. A, B, C and D different superscripts within each column show significant differences at the level of MDA between the females of each group. a, b, c and d different superscripts within each column show significant differences at the level of MDA between the males of each group.

The levels of proteins, reduced glutathione (GSH) and lipid peroxidation (MDA) in plasma of the studied animals are summarized in Figs. 3, 4 and 5. All these parameters were not significantly influenced by the sex (*p* > 0.05). In both males and females, significant (*p* < 0.0001) difference in plasma protein, GSH and MDA levels was recorded between all the studied groups.

**Figure 6:**
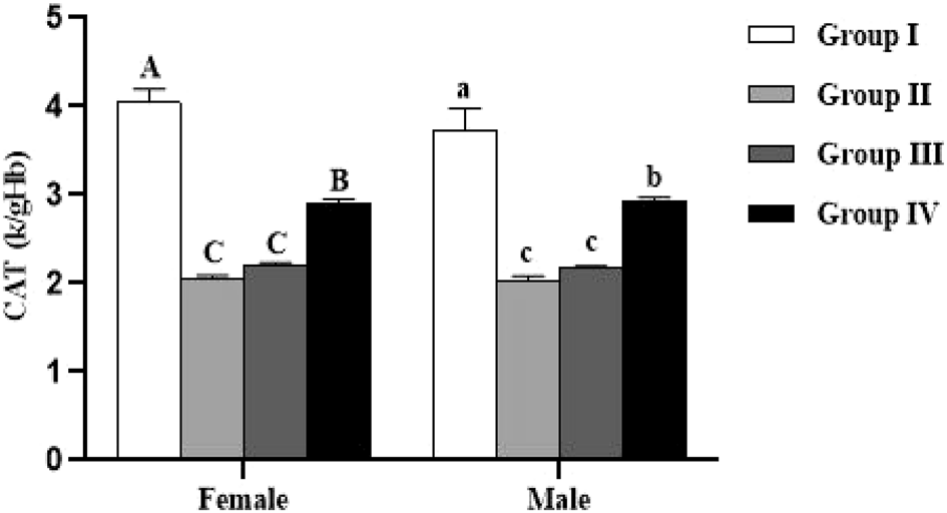
Effect of *Spirulina platensis* on catalase (CAT) activity in lambs reared in endemic fluorosis area. Data were expressed as the mean ± SEM. Group I: lambs reared in fluorosis-free area, Group II: lambs reared in endemic fluorosis area, Group III: lambs supplemented with 250 mg/kg BW/day of *Spirulina platensis*, Group IV: lambs supplemented with 500 mg/kg BW/day of *Spirulina platensis*. A, B, C and D different superscripts within each column show significant differences at the level of CAT activity between the females of each group. a, b, c and d different superscripts within each column show significant differences at the level of CAT activity between the males of each group.

**Figure 7:**
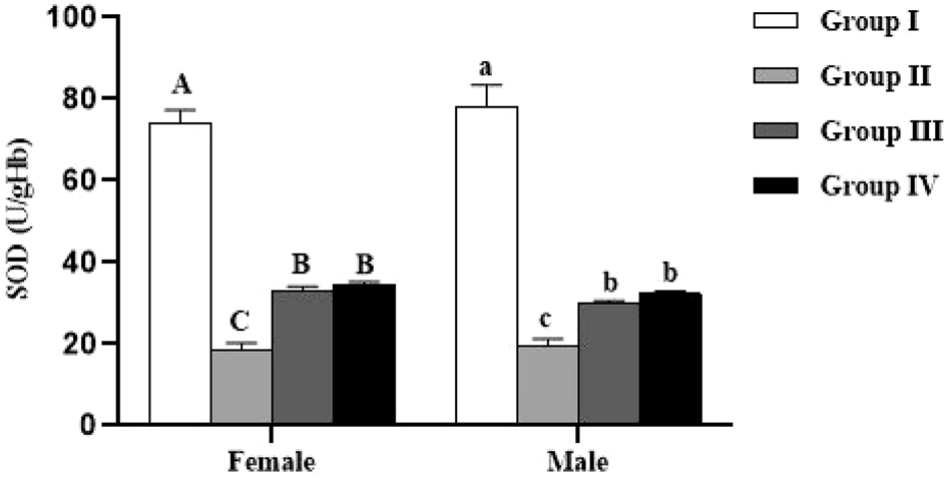
Effect of *Spirulina platensis* on superoxide dismutase (SOD) activity in lambs reared in endemic fluorosis area. Data were expressed as the mean ± SEM. Group I: lambs reared in fluorosis-free area, Group II: lambs reared in endemic fluorosis area, Group III: lambs supplemented with 250 mg/kg BW/day of *Spirulina platensis*, Group IV: lambs supplemented with 500 mg/kg BW/day of *Spirulina platensis*. A, B, C and D different superscripts within each column show significant differences at the level of SOD activity between the females of each group. a, b, c and d different superscripts within each column show significant differences at the level of SOD activity between the males of each group.

Concerning enzymatic activity, the results showed that catalase and SOD activities were significantly lower in female (*p* < 0.0001) and male (*p* < 0.0001) lambs reared in endemic fluorosis area (Group II) compared to those reared in fluorosis-free area (Group I) (Figs. 6 and 7). Moreover, in both male and female lambs supplemented with 500 mg/kg BW/day of *Spirulina platensis* (Group IV), it was revealed that catalase (CAT) and superoxide dismutase (SOD) are significantly lower (*p* < 0.0001) compared to Group I and significantly higher (*p* < 0.0001) compared to Group II (Figs. 6 and 7). Concerning the group supplemented with 250 mg/kg BW/day of *Spirulina platensis* (Group III), the results revealed that there was no significant difference (*p* > 0.05) in catalase activity in both sexes compared to the Group II, whereas SOD activity was significantly lower (*p* < 0.0001) than Group I and significantly higher than Group II (*p* < 0.0001).

**Figure 8:**
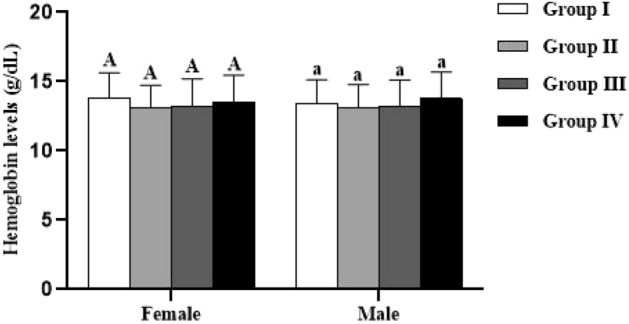
Effect of *Spirulina platensis* on hemoglobin (Hb) levels in lambs reared in endemic fluorosis area. Data were expressed as the mean ± SEM. Group I: lambs reared in fluorosis-free area, Group II: lambs reared in endemic fluorosis area, Group III: lambs supplemented with 250 mg/kg BW/day of *Spirulina platensis*, Group IV: lambs supplemented with 500 mg/kg BW/day of *Spirulina platensis*. A: indicates that there is no significant difference in hemoglobin levels between the females of each group. a indicates that there is no significant difference in hemoglobin levels between the males of each group.

Coming back to the level of hemoglobin, the results of Fig. 8 indicated that this parameter was not significantly affected by either the studied groups or sex (*p* > 0.05).

According to the Rho Spearman test (Table 3), the results revealed that plasma fluoride level were positively correlated with GSH (r = 0.18; *p* < 0.0001) and MDA (r = 0.19; *p* < 0.0001), and negatively correlated with proteins (r = − 0.14; *p* < 0.0001), catalase (r = − 0.11; *p* < 0.0001) and SOD (r = − 0.22; *p* < 0.0001).

**Table 3:** Correlation between fluoride, protein, MDA, GSH levels and catalase and SOD activities.

	Fluoride	Proteins	GSH	MDA	Catalase	SOD
Fluoride	1	− 0.14*	0.18*	0.19*	− 0.11*	− 0.22*
Proteins	− 0.14*	1	− 0.24*	− 0.15*	0.22*	0.11*
GSH	0.18*	− 0.24*	1	0.55*	− 0.58*	− 0.38*
MDA	0.19*	− 0.15*	0.55*	1	− 0.39*	− 0.37*
Catalase	− 0.11*	0.22*	− 0.58*	− 0.39*	1	0.36*
SOD	− 0.22*	0.11*	− 0.38*	− 0.37*	0.36*	1

*Correlation is significant at the 0.0001 level.

## Discussion

This study investigated the effects of the natural microalgae called *Spirulina platensis* supplemented to lambs reared in El fokra commune, belonging to Khouribga-Morocco where fluorosis is endemic. In adherence to the null hypothesis, which postulated that there would be no significant protective effect of *Spirulina platensis* supplementation at two different doses (250 and 500 mg/kg BW/day) against dental fluorosis and oxidative stress in lambs, the present study was conducted. However, the findings lead us to reject the null hypothesis.

In Morocco, two distinct types of fluorosis are recognized, industrial and hydrotelluric^28^. However, the predominant form in Morocco is hydrotelluric fluorosis, mainly affecting areas rich in phosphate^29^, notably the province of Khouribga^17^. These regions are home to natural deposits of fluoride within phosphate rocks, and weathering of these rocks releases a significant amount of fluoride into groundwater and soil^23^.

Our study indicates significantly elevated fluoride levels in soils and groundwater in the commune of El Fokra compared to the Settat region, confirming the prevalence of hydrotelluric fluorosis in the former area. Furthermore, another study revealed higher fluoride concentrations in plant species (*Chrysanthemum coronarium*, *Hordeum vulgare*, *Papaver rhoeas*, *Reseda alba*, *Silene vulgaris*, *Sinapis alba* and *Fumaria parviflora*) collected in the grazing areas of El Fokra, as opposed to Settat^30^. This suggests that consumption of water or plants grown in fluoride-contaminated soil by lambs reared in El Fokra commune can lead to various forms of fluorosis.

The results obtained showed that the lambs reared in this commune presented dental lesions with a score varying between very mild to moderate according to Dean’s Index (DI). Several studies have shown that fluorosis in sheep, whether endemic or experimentally induced, results in dental lesions of varying severity depending on the concentration and the daily amount of ingested fluoride^14,31–33^. Daily intake of 250 mg/kg BW/day showed an ameliorative effect by reducing the degree of severity of dental lesions, while 500 mg/kg BW/day of *Spirulina platensis* protected against dental fluorosis in lambs. Rahim et al.^27^ reported that *Spirulina platensis* used in this study contains high levels of certain minerals, including calcium, magnesium, manganese, and zinc. These minerals have a strong affinity to fluoride, which means they can bind to it and reduce its harmful effects^34^. According to other studies, the high levels of calcium and ascorbic acid naturally present in plants, grass, and forage were proven to be responsible for the lower prevalence of dental and bone lesions in sheep reared in fluorosis endemic areas^35,36^.

Higher plasma fluoride level in fluorotic lambs was observed in this study compared to healthy control. Several studies reported that supplementing animals with some synthetic minerals reduced plasma fluoride in sheep^21,22^. This study was the first to evaluate the effect of a natural microalgae (*Spirulina platensis*) in lambs. Daily intake of 500 mg/kg BW of this microalgae reduced the plasma fluoride level by about 67.51%. On the other hand, the drop of plasma protein level in fluorotic animals in this study was similar to other findings^37,38^. In this regard, it was reported that fluoride can trigger endoplasmic reticulum stress, which in turn can reduce protein synthesis and secretion^39^. Moreover, DenBesten and Li^40^ reported that the inhibition of protein synthesis produced by fluoride toxicity during enamel development was responsible for dental fluorosis in the incisors of the animals. Hence, the beneficial effect of *Spirulina platensis* in increasing the level of plasma proteins in this work can be explained by its high crude protein content^41,42^. The term crude proteins refer to the total protein content in *Spirulina platensis* without further refinement or processing, a standard metric in nutritional analysis encompassing all forms of proteins without distinguishing types or quality. *Spirulina platensis*, used in this research, contains 53.31 g of proteins per 100 g of the microalgae, including essential amino acids and various protein compounds^27^. This substantial presence of crude proteins holds significance as it suggests *Spirulina platensis* may contribute to overall protein intake in animals. This aspect potentially plays a role in mitigating the adverse effects of fluoride toxicity on protein synthesis^43,44^. Furthermore, the findings of the current study revealed that the fluoride excretion level via feces was the same in all studied groups. This could be explained by the fact that the main route of fluoride excretion is urination (50–70%)^20^.

Regarding antioxidant system parameters, the results indicated that the erythrocytic enzymatic activities of CAT and SOD were lower while plasma levels of GSH and MDA were higher in fluorotic lambs compared to the healthy control. From the previous studies, it has been shown that fluoride is related to oxidative stress^45–47^. Moreover, it has been suggested that there are many groups of enzymes that can be affected by fluoride. More precisely, several enzymes require divalent metal ions as cofactors such as magnesium, calcium, zinc and selenium which have a strong affinity with fluoride, and thus can induce oxidative stress resulting from an inhibitory interaction of these enzymes^13,48,49^. For that, recent studies focused on supplementation of animals with antioxidants such as gallic acid^50^, quercetin^51^, vitamins^7,46,52^ and medicinal plants^6,19,26^. In this study, *Spirulina platensis* showed an ameliorative effect against oxidative stress in fluorotic sheep species. The ability of *Spirulina platensis* to prevent lipid peroxidation through chronic fluoride ingestion could be linked to its arsenal of antioxidants including phenolic compounds, β-carotene, vitamins and chlorophyll^53^. It also contains several natural phycobiliproteins, mainly phycocyanin which is a hydrophilic and intense blue-colored molecule, having a linear tetrapyrol prosthetic group called bilin. The latter gives it a strong ability to scavenge radical oxygen species and can behave as an antioxidant^54^. Additionally, phycocyanin originated from *Spirulina platensis* contains high levels of glutamic acid, glycine, and cysteine which are precursors of glutathione^55^. The latter is a powerful antioxidant that protect cells from oxidative damage. β-carotene, zeaxanthin, lycopene, lutein and chlorophyll which are present in *Spirulina platensis* have received considerable attention, due to their ability to strengthen the physiological system against oxidative stress^53,56^. These molecules can directly scavenge free radicals and inhibit lipid peroxidation, or indirectly improve the enzymatic activity of SOD and catalase^57,58^. Liu et al.^59^ observed a significant inverse association of the skeletal fluorosis with dietary intake of β-carotene, lutein/zeaxanthin, lycopene, and total carotenoids. Oxidative stress has been shown to play a potential etiological function through the expression and signalling process of NF-κB ligand (RANKL), which enhances osteoclast genesis and promotes osteoclastic differentiation in bone loss^60,61^. On the other hand, lycopene may exert dual anti-catabolic and pro-anabolic activities in bone by counteracting the NF-κB signal transduction pathway, inhibiting osteoclast differentiation and promoting osteoblast mineralization^62^. Moreover, it has been proposed that lycopene has a beneficial impact on bone formation and may imply possible consequences due to its use in the chemoprevention of skeletal fluorosis^59^.

Coming back to other components in *Spirulina platensis* that could have a main role in explaining our findings, it has to be highlighted that this microalga is a rich source of natural acidic polyphenols^63^. The most abundant polyphenols in *Spirulina platensis* are gallic acid and caffeic acid^64^, histopathological studies have shown that caffeic acid protects against fluoride-induced liver damage, and decreases serum fluoride levels and regulates the level of enzymatic and non-enzymatic antioxidants. In addition, caffeic acid inhibits fluoride induced apoptosis by altering the expression of the Bax and caspase-3p20 genes^65^. Another study showed that gallic acid can attenuate nephrotoxicity and oxidative stress induced by fluoride^51^. It also protected against fluoride-induced hypertension and hepatorenal toxicity by lowering blood pressure, inhibiting lipid peroxidation, protein carbonylation, and enhancing the enzymatic and non-enzymatic antioxidant pathways^66^. Further, and more importantly, to minimize the cost of *Spirulina platensis*, several recent studies have been focused on the culture of this microalgae in natural and cheaper mediums instead of the standard Zarrouk^67^ which is synthetic and expensive^68–71^. Accordingly, *Spirulina platensis* used in this study is cultivated using a natural and cheap medium based on well water and wood ash^27^. This medium allowed the production of a biomass like the Zarrouk medium. Furthermore, it increased the production of antioxidants such as phycocyanin, carotenoids and water-soluble vitamins mainly ascorbic acid^27^.

This study indeed provides valuable insights into the potential protective effects of *Spirulina platensis* against dental fluorosis and oxidative stress in naturally exposed lambs, rejecting the null hypothesis. The importance of extrapolating these findings to human contexts is acknowledged. While animal studies like this one serve as essential preliminary investigations, it is crucial to recognize the limitations and the need for cautious interpretation. Firstly, the small sample size in the study is indeed a valid point. However, it is essential to clarify that this animal study was conducted as a Phase I interventional study. Such studies often start with small sample sizes to assess safety, feasibility, and preliminary efficacy trends before larger, more generalizable trials are undertaken. The decision to employ a small sample size was not arbitrary but rather a deliberate choice made to ensure the ethical use of animals and minimize harm while providing a foundation for future research. Regarding statistical analysis, the study was conducted with a focus on achieving a high statistical power, set at 95%. This choice was based on the acceptable level of type II error (false negatives), ensuring a strong likelihood of detecting true differences if existed. The choice of Fisher’s exact test for analyzing categorical data with small sample sizes was motivated by the suitability of this method for such scenarios. To sum up, while this study does have limitations, including the small sample size and specificity to a particular geographic area, it is essential to view these limitations within the context of the study’s purpose and design. This animal study serves as an initial step in exploring the potential benefits of *Spirulina platensis* in reducing dental fluorosis and oxidative stress. Further research, including larger-scale studies and human trials, will be necessary to validate and generalize the findings to broader populations.

## Conclusion

In conclusion, this interventional clinical trial provides strong evidence supporting the efficacy of *Spirulina platensis* supplementation in reducing dental fluorosis severity and improving oxidative stress markers in lambs naturally exposed to fluoride. These results directly concern the central questions of our study. While the potential for broader applications of *Spirulina platensis* in addressing fluorosis in other animal populations and potentially in human contexts is an area of interest, it is important to emphasize that our conclusions are firmly based on the evidence obtained in this specific research study. Further investigation, including Phase I/II trials, is warranted to explore the practical implications of *Spirulina platensis* in managing fluorosis in diverse and varied environments.

## Methods

### Microalgae material

*Spirulina platensis* was purchased from an artisanal farm situated at Chichaoua-Morocco (31° 32′ 38″ north, 8° 45′ 58″ west). It was cultivated in natural climatic conditions and wholly characterized by Rahim et al.^27^.

### Study site

This study was conducted in El Fokra commune, located in the western region of Khouribga, Morocco (32° 53′ 9.683″ N, 6° 55′ 15.116″ W). El Fokra commune is a part of the phosphate zone, characterized by geological conditions that contribute to the prevalence of hydrotelluric fluorosis in the area^30^. Hydrotelluric fluorosis indicates that the two main sources of contamination in this region are water and soil. The region’s semi-arid climate, marked by hot and dry summers, leads to elevated evaporation rates, potentially impacting fluoride concentrations in local water sources, soil, and vegetation, and influencing the health and behavior of the animal subjects. For comparison, lambs from the fluorosis-free Settat region, located nearby (33° 0′ 0″ N, 7° 37′ 0.12″W), were also included in the study.

### Collection of groundwater and soil samples

A total of 20 well water samples were collected, with 20 samples originating from the El Fokra commune and 20 samples from the Settat region. These wells had depths ranging between 60 and 80 m, corresponding to the depth at which water is typically accessed from the groundwater table. Each water sample, approximately 100 mL in volume, was carefully collected in clean and sterile polyethylene bottles, and stored at 4 °C until analyzed.

Soil samples were collected from various locations in both the El Fokra and Settat regions, with 20 samples gathered from each location. Each soil sample was carefully placed in clean and sterile polyethylene bottles and transported to the laboratory. Subsequently, the soil samples were subjected to a drying process in an oven at 105 °C for 24 h and then ground into a fine powder using a mortar and pestle. For the digestion process, 1 g of powdered soil from each sample was placed in a crucible, covered with 5 mL of 8 M NaOH solution, and heated on a hot plate for a minimum of 5 min. The crucibles were then placed in a 200 °C oven for 16 h. After cooling, the crucibles were rehydrated with 15 mL of distilled water, gently shaken until homogeneous, and neutralized to pH 7.2–7.5 using 37% hydrochloric acid. Finally, the solutions were diluted with 25 mL of distilled water, and stored until analyzed^72^.

### Animals and experimental design

All animal care protocols were accomplished in agreement with the Hassan II Institute of Agronomy and Veterinary Medicine of Rabat and the Moroccan Ministry of Agriculture recommendations which are in accordance with international ethical standards (European Union Directive 2010/63/EU) legislation and ARRIVE (Animal Research Reporting of In Vivo Experiments) guidelines.

A total number of forty-eight Sardi lambs aged 5 months were chosen for the present experiment. This work was conducted as a Phase I interventional study, which typically starts with small sample sizes to assess safety, feasibility, and preliminary efficacy trends before larger trials. This choice aimed to ensure ethical use of animals and minimize harm while providing a foundation for future research. The decision to employ a small sample size was deliberate and not arbitrary. It was made to address ethical concerns and align with the study’s initial goals. Practical considerations, including the availability of lambs meeting specific criteria and limitations in available resources (time, personnel, budget), influenced the final sample size choice. To reinforce the reliability of our findings, we utilized an online sample size calculator, setting a desired statistical power of 95% and a significance level of 0.05. This approach ensured a 95% chance of detecting true differences if they existed while maintaining a 5% threshold for the risk of type I errors, aligning with common standards in scientific research.

**Figure 9:**
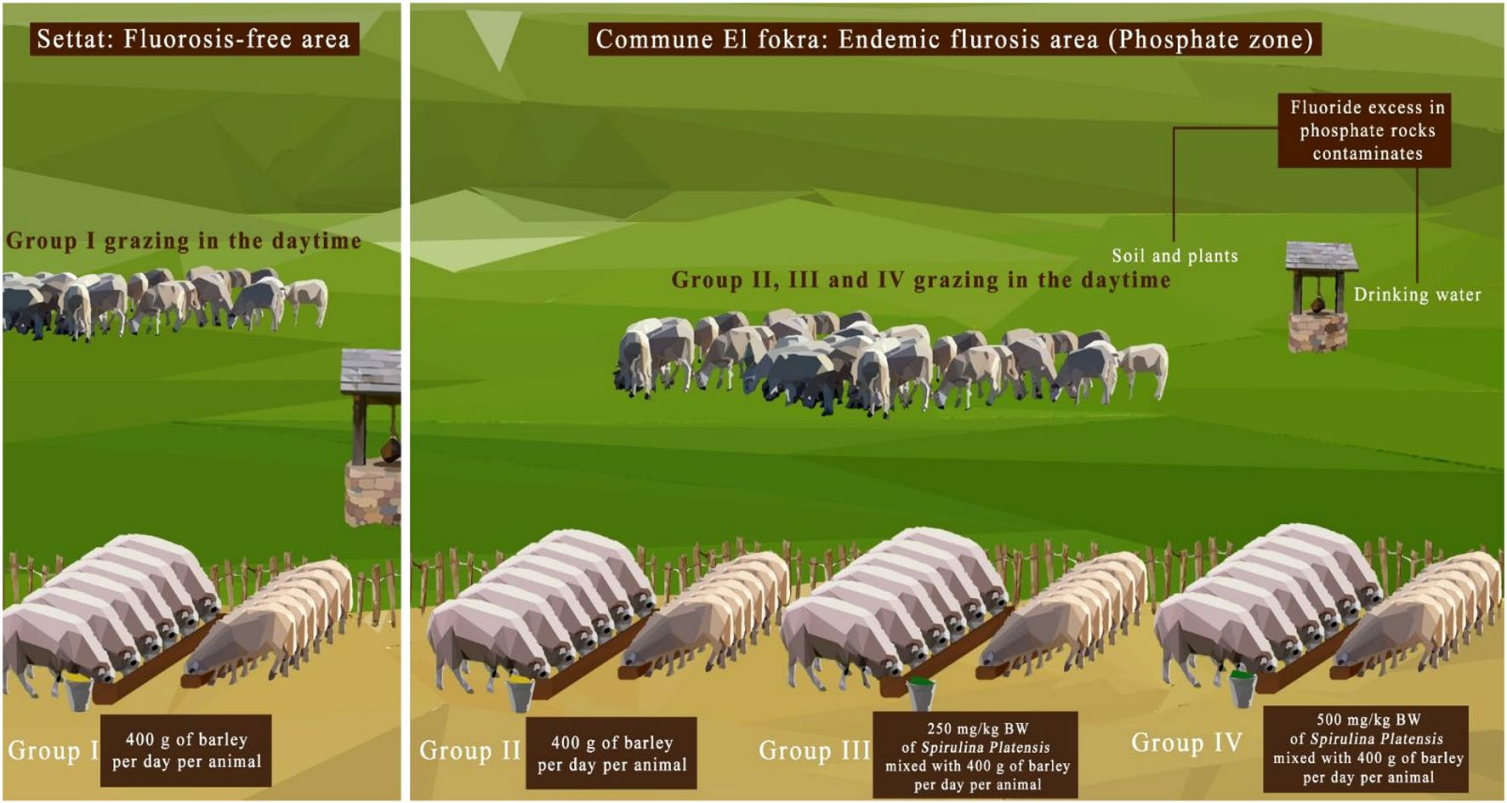
Diagram presenting the experimental protocol.

They animals were weighed and then treated against enterotoxaemia and internal and external parasites. The experiment started when the lambs were 5 months old and ended in August 2021 after the eruption of their first two milk teeth and the total appearance of their incisors. Twelve lambs reared at INRA belonging to the Regional Agricultural Research Center station in Settat city, were used as a control of a fluorosis-free area and then used as healthy control (Group I). The Thirty-six animals reared in the endemic fluorosis (El fokra-Khouribga) area were separated into 3 equal groups (II, III and IV). Each group was composed of 6 females and 6 males (Fig. 9).

- Group I: healthy control reared in a fluorosis-free area and each animal received every morning 400 g of barley without *Spirulina platensis*.
- Group II: reared in endemic fluorosis area and each animal received every morning 400 g of barley without *Spirulina platensis*.
- Group III: received every morning 250 mg/kg BW of *Spirulina platensis* mixed with 400 g of barley, for each animal.
- Group IV: received every morning 500 mg/kg BW of *Spirulina platensis* mixed with 400 g of barley, for each animal.

All animals were allowed to be taken to pasture and reared extensively. Groups III and IV were weighed every 15 days to readjust the quantity of *Spirulina platensis* according to BW of the animal.

### Dental examination

After the eruption of their two first milk teeth, lambs were clinically examined for lesions on their incisors. Dental lesions were scored according to the Dean’s Fluorosis Index (DFI)^73^. This index based on six scores attributed to tooth damage from fluorosis^23^.

### Collection of blood samples and feces

The samples of blood were collected every 15 days from the jugular vein in heparinized tubes. Then the samples were centrifuged at 3000 g/10 min at 4 °C, and the supernatant containing plasma was stored at − 20 °C until use. Besides, the pellet containing the erythrocytes was washed by NaCl (9 g/L) three times to eliminate contaminants before being stored at − 20 °C until use.

Concerning feces samples, a thick filter paper was used to catch the feces from each group of animals. Afterward, the obtained feces were dried at 100 °C and grounded with a mortar. Then, the dried and powdered fecal samples underwent digestion using the same method employed for soil samples^72^, and stored until analyzed.

### Determination of fluoride in plasma and feces

To determine the level of plasma fluoride, 1 mL of plasma of each animal was buffered with 1 mL of the commercial total ionic strength adjustment buffer II solution (Orion 940,909; Thermo Fisher Scientific, Waltham, MA, USA) and evaluated using a fluoride electrode (Orion 96-09; Thermo Fisher Scientific, Waltham, MA, USA), coupled to an ion analyzer (Star A214; Thermo Fisher Scientific, Waltham, MA, USA). The electrode was calibrated with standard fluoride solutions (Orion 940,907; Thermo Fisher Scientific, Waltham, MA, USA).

### Biochemical parameters

A total number of 1500 blood samples collected during 13 months of the experiment underwent various biochemical analyzes to judge the effect of *Spirulina platensis*.

### Reduced glutathione level

Reduced glutathione (GSH) level in plasma was determined according to Ellman^74^. Briefly, 200 µL of trichloroacetic acid 5% was mixed with 400 µL of plasma. After centrifugation for 10 min at 12,000 g, 50 µL of supernatant was mixed with 850 µL of phosphate buffer (50 mM, pH 8) and 100 µL of 6 mM 5,5-dithiobis (2-nitrobenzoic acid (DTNB)). The yellow-colored solution was immediately read at 412 nm, using a Shimadzu UV-1601 Spectrophotometer (Shimadzu; Kyoto, Japan). Glutathione reduced was used as a standard.

### Malondialdehyde level

Malondialdehyde level was measured according to the standard method^75^. Briefly, 1.5 mL of TBA (0.8%) was added to 1 mL of the plasma of each animal. Then 0.4 mL of SDS (8.1%) and 1.5 mL of acetic acid were added. The mixture was finally made up to 5 mL with distilled water and placed in hot water bath at 95 °C for 1 H. After cooling 1 mL of distilled water and 5 mL of n-butanol was added. The mixture was vortexed and after centrifugation for 10 min at 4000 g, the absorbance of the organic layer (upper layer) was measured at 532 nm by a Shimadzu UV-1601 Spectrophotometer (Shimadzu; Kyoto, Japan).

### Assays of antioxidant enzymes

Catalase activity was determined by the method developed by Ni et al.^76^. The 200 µL reaction mixture containing 10 µL of the aliquot was mixed with 30 µL of a 7.3 mM H_2_O_2_ solution. After 3 min of incubation, the reaction was stopped by adding 20 µL of H_2_SO_4_ at 6N and then 140 µL of KmNO_4_ at 2 mM. The absorbance was read at 480 nm by a Tecan Microplate Reader Infinite 200 Pro (Tecan; Männedorf, Switzerland). The specific activity of CAT was expressed as micromoles per minute per milligram of protein. Superoxide dismutase (SOD) activity was assayed as described by Beyer and Fridovich^77^. The reaction mixture contained 50 mM phosphate buffer, 0.025% Triton X-100, 0.1 mM EDTA (pH 8), 12 mM L-methionine, 75 mM NBT, aliquot and 2 µM riboflavin. The tubes were shaken and placed 30 cm below a light bank consisting of a 15 W fluorescent lamp for 10 min. The reaction was stopped by switching off the light and the absorbance was read at 560 nm by a Tecan Microplate Reader Infinite 200 Pro (Tecan; Männedorf, Switzerland).

### Proteins and hemoglobin levels

Plasma proteins were estimated using the BCA Protein Assay Kit^78^. Hemoglobin (Hb) concentrations were determined using drabkin’s cyanmethemoglobin method^79^. All samples were analyzed in triplicates.

### Statistical analysis

To determine the appropriate sample size, we utilized the online tool available at ‘https://clincalc.com/stats/samplesize.aspx’. The desired statistical power represents the probability of correctly detecting a true difference if it exists. It was chosen based on the acceptable level of type II error (false negatives) and was set at 95%. The significance level was set at 0.05. To compare the dentition among the four groups, we used the R programming language and specifically, the fisher test function. Fisher’s exact test was chosen due to its suitability for analyzing categorical data when comparing multiple groups with small sample sizes. The null hypothesis assumed no association between the dentition differences and the groups, while the alternative hypothesis suggested a significant association. Additionally, a comparison of means for plasma fluoride, fecal fluoride, and biochemical parameters between groups and sexes was performed using JMP SAS (SAS Institute; Cary, NC, USA). The Wilcoxon test was employed as the statistical method in this analysis.

## Data Availability

The datasets used and/or analyzed during the current study available from the corresponding author on reasonable request.
